# Families’ importance in nursing care–families’ opinions: a cross-sectional survey study in the homecare setting

**DOI:** 10.1186/s13690-024-01314-4

**Published:** 2024-06-17

**Authors:** Josien M. Woldring, Wolter Paans, Reinold Gans, Laura Dorland, Marie Louise Luttik

**Affiliations:** 1 Research Group Nursing Diagnostics, Family Care & Family Nursing, School of Nursing, Hanze University of Applied Sciences, Petrus Driessenstraat 3, Groningen, 9714 CA The Netherlands; 2 Department of Internal Medicine, University Medical Center Groningen, University of Groningen, Hanzeplein 1, Groningen, 9713 GZ The Netherlands; 3 Department of Critical Care, University Medical Centre Groningen, PO Box 30.001, Groningen, 9700 RB The Netherlands; 4 Merkbaar Beter, PO Box 102, Espria, Beilen, 9410 AC the Netherlands

**Keywords:** Collaboration, Division of care, Family opinion, Family perspective, Healthcare professionals, Homecare services, Home nursing, Informal care, Nursing care

## Abstract

**Background:**

Informal care is an essential part of support provided in the homecare setting. To ensure effective healthcare provision, good communication and collaboration between informal and formal care providers are crucial. To achieve this aim, it is necessary to have a clear understanding of the perspectives of all stakeholders. In the scientific literature, limited knowledge is available regarding family members’ opinions about their involvement in care. To date, no instruments have been developed that accurately measure these opinions. This study aims to elucidate the opinions of family members about their involvement in nursing care.

**Methods:**

A cross-sectional survey approach was employed. The methodological steps in this study were (1) convert the Families’ Importance in Nursing Care–Nurses’ Attitudes (FINC-NA) from a nurses’ perspective to a family perspective and thus develop the Families’ Importance in Nursing Care–Families’ Opinions (FINC-FO) and (2) measure families’ opinions regarding their involvement in home nursing care. The questionnaire was sent to 3,800 patients with activated patient portals, which accounts for about 17% of the total patient base. Responses were received from 1,339 family members, a response rate of 35%.

**Results:**

The developed FINC-FO questionnaire showed homogeneity and internal consistency. The results of the questionnaire indicate that family members consider it important to be involved in care and that they wish to be acknowledged as participants in discussions about care (planning) but are less inclined to actively participate in the provision of care by nurses. Family members expressed less explicit opinions about their own support needs. Factors such as level of education, type of partnership, and amount of care provided are seemingly associated with these opinions.

**Conclusions:**

Family members in the homecare setting wish to be involved in discussions about care (planning). The transition in care from primarily formal to more informal care necessitates an awareness and clear definition—on part of both healthcare professionals and families—of their respective roles in the provision of care. Communication about wishes, expectations, and the need for support in care is essential to ensure quality of care and that the family can sustain caregiving.

**Table:** 

**Contributions to the literature**
• Limited knowledge is available regarding family members’ opinions about their involvement in care, and to date, no instruments have been developed that accurately measure these opinions.
• The developed FINC-FO seems to be a feasible questionnaire to measure families’ opinions about their involvement in nursing care in the home setting.
• This study offers insight into family members’ opinions on their involvement in caregiving and the influencing factors. It underscores the importance for both healthcare professionals and families to cultivate awareness and establish clear definitions of their respective roles in providing care.

## Background

Informal family care is an essential aspect of healthcare that involves the provision of support to family members who are ill, disabled, or vulnerable [1]. Family caregivers (e.g., partner, child, neighbor, friend) are vital for patients’ support and informal care [2, 3]. In recent years, the need for support from family caregivers at home has increased due to societal changes, such as the aging population and the decreasing availability of institutionalized professional care for daily support. As a result, vulnerable, dependent elderly people continue to live at home for longer periods but are less able to rely on professional care. These societal changes necessitate an appropriate transition from primarily formal to more informal care. Informal care provided by family benefits patients’ wellbeing; however, it is also associated with a range of practical, physical, and emotional challenges for family members [4, 5].

To make this transition of care successful, a need exists for targeted, effective communication that facilitates collaboration between healthcare professionals and informal family caregivers. Healthcare professionals should view family caregivers as partners in the care process to meet patient and family needs [6]. Earlier research indicates that preparedness for caregiving depends on the support that families receive from healthcare professionals [7]. To achieve good communication and collaboration between healthcare professionals and family caregivers, it is important to know both families’ and healthcare professionals’ opinions regarding the role of family members in caring for patients. Earlier research further indicates that nurses who generally have positive attitudes toward involving families as partners in patient care are more likely to communicate and collaborate with families [8]. With the increasing importance of family caregivers at home, it is implicitly expected that in general, family members wish to be involved in care. However, limited research has been conducted on families’ opinions regarding their involvement in direct nursing care and, subsequently, how they prefer to communicate and collaborate with nurses [9]. Involvement in the care for a family member is likely to be imagined differently by and between family members, which may differ from what is expected by nurses [10, 11]. It is thus crucial to understand the opinions of family members regarding their involvement in nursing care and determine whether families’ wishes and expectations align with the principles of care envisioned by nurses. As such, this study aims to explore family members’ opinions regarding their involvement in nursing care for relatives in a homecare setting.

## Methods

Instruments exploring family members’ opinions regarding their involvement in nursing care are currently lacking. As such, we have adapted the widely used Families’ Importance in Nursing Care–Nurses’ Attitudes (FINC-NA) from a nursing to a family perspective.

The methodological steps employed in this study were to (1) convert FINC-NA from a nursing to a family perspective and thus develop the Families’ Importance in Nursing Care–Families’ Opinions (FINC-FO) and (2) measure families’ opinions regarding their involvement in nursing care at home. A cross-sectional survey approach adhering to the “Strengthening the Reporting of Observational Studies in Epidemiology” guidelines for articles reporting cross-sectional studies was employed [12].

### Instrument

FINC-NA, a widely used instrument to measure nurses’ attitudes toward the importance of involving families in nursing care [13, 14], is based on family systems nursing theory and has been validated in different healthcare settings and countries [15, 16]. The study of Hagedoorn et al. (2018) provides an overview of countries that have validated the FINC-NA. Examples of nurses and care settings described in this study are registered nurses in Sweden, psychiatric nurses in Iceland and Taiwan, primary healthcare nurses in Portugal and hospital nurses in Portugal and Australia [16]. In educational attainment, there are slight variations, but al nurses maintain an educational level comparable to registered nurses.

The FINC-NA comprises four subscales: *family as a resource in nursing care*, referring to a positive attitude toward families’ presence in nursing care; *family as a conversational partner*, referring to the acknowledgment of patients’ families as conversational partners; *family as a burden*, referring to statements of experiencing family as a burden; and *family as its own resource*, referring to families’ own resources for coping [17]. Other instruments exist that measure families’ perspectives toward family involvement, but these typically involve a specific context and focus on families’ experiences with care, rather than their opinions about how they want to be involved. As such, we have adapted FINC-NA from a nursing to a family perspective.

#### Converting the survey items

The FINC-NA questionnaire has undergone translation into Dutch and subsequent psychometric testing. Hagedoorn et al. (2018) details this linguistic validation process, which involved translating the original Swedish questionnaire to Dutch [16]. This Dutch version of FINC-NA, comprising 26 statements utilizing a 5-point Likert scale, was converted from a nursing to a family perspective, resulting in the Dutch FINC-FO. To remain as close as possible to the original statements of the validated list, initially, only the concept of “nurse” was converted to that of “family” (or vice versa). The statements were subsequently evaluated and adjusted by two researchers (MLL and LD) with expertise in family care, who aimed to maintain the intention of the statements while ensuring that they were also easily understandable and applicable from a family perspective. Most of the adjustments involved the addition of the words “I consider it important…”. Only one item from FINC-NA, on the subscale *family as resource in nursing care* (“The presence of families gives me a feeling of security”), could not be transferred to a family perspective. As this statement relates to nurses’ emotions and perceptions, it was not considered something that the family could express an opinion on.

The content validity of the FINC-FO items was established by homecare patients and experienced informal caregivers (4 in total) who were members of an official customer council within a homecare organization. The FINC-FO was sent to the council by e-mail. Members of the council were asked to review all 26 statements for clarity and relevance and provided written feedback to the research team. Some statements were assessed as unclear, which could subsequently be resolved by changes in word-order or word-choice. All experts agreed that the statements in the final version of FINC-FO were clear and relevant to examine families’ opinions about their involvement in care at home.

The converted questionnaire resulted in an FINC-FO list comprising 25 statements exploring the four subscales using a 5-point Likert scale (1 = strongly disagree–5 = strongly agree) aligning with the original FINC-NA. Items were presented per subscale, starting with *family as a resource in nursing care*, with nine items, followed by *family as a conversational partner*, with eight items, then *family as a burden*, with four items, and finally *family as its own resource*, also comprising four items [12].

#### Reliability and construct validity

An item-total correlation correcting for overlap was conducted to evaluate the homogeneity and discrimination ability of the items. This correlation should be higher than 0.30 [18]. Cronbach’s alpha was used as a measure of the internal consistency or reliability of FINC-FO and its subscales. An alpha value of 0.70 or higher is generally considered acceptable, while values of 0.80 or higher are considered excellent [18]. To analyze the questionnaire’s construct validity, a confirmatory factor analysis was used. Since FINC-FO is based on the theory of FINC-NA, a deductive theory-based approach with the original, pre-specified factor structure of the four constructs was tested. A one-factor analysis per subscale was used to investigate the size of loadings (i.e., the items’ degree of association with the latent factor). Stevens (2002) recommends interpreting factor loadings with absolute values above 0.40 as sufficient [19].

### Sample and setting

FINC-FO was distributed among the family members of patients receiving care from three home healthcare organizations in the northern region of the Netherlands. In the Dutch research context, homecare institutions are defined as organizations that deliver varying levels of care within individuals’ homes, serving to different levels of complexity. These organizations match the complexity of care required with the training and competency level of the healthcare professional, typically categorized by nursing levels. This coordination is facilitated through a nursing assessment performed by registered nurses. This model of healthcare organization is generally analogous to home healthcare organization in other European countries and North America.

As the FINC-FO questionnaire was made available exclusively through the electronic health record system’s patient portal (Caren-Nedap), only family members admitted to the patient portal were able to participate. The questionnaire was sent to 3,800 patients with activated patient portals, which accounts for about 17% of the total patient base. Responses were received from 1,339 family members, a response rate of 35%.

### Data collection

In April 2022, FINC-FO was administered to family caregivers through the patients’ electronic health record system. The questionnaire was accessible via a link within the patient’s care file, visible to both patients and their families. Demographic characteristics such as age, gender, level of education, type of relationship with the patient, number of hours of caregiving, and working status were subsequently collected. The entire data collection process took 4 weeks.

### Data analysis

Only completed questionnaires were included in the data analysis. Of the 1,339 questionnaires, 64 were excluded due to incomplete responses, resulting in 1,275 questionnaires for data analysis. As in FINC-NA, items on the subscale *family as a burden* were reverse scored, so the scores on this scale were recoded before analysis. Education level was categorized as high (tertiary education), middle (secondary education), or low (primary education), and the categories of relationship to the patient were merged into three: spouse, parent/child, and other. Data were analyzed using SPSS for Windows (release 28.0.1.1), and descriptive statistics were used to describe the study population and the responses to the FINC-FO questionnaire on item levels. Higher scores indicate more positive opinions. An independent t-test and an ANOVA were used to compare differences in attitudes related to background variables. For these analyses, the continuous variables age and caregiving hours were dichotomized. Mean or median was used as the cut-off point for the distribution. Multivariable linear regression analyses were performed to determine the individual contribution of each background variable to the FINC-FO and subscale scores. The significance level was set at *p* ≤ 0.05.

## Results

### FINC-FO questionnaire

The questionnaire was completed by 1,275 respondents. Table 1 illustrates the subscales, with the associated FINC-FO items. Subscales and items are shown in the same order as they appear in the questionnaire. All items on the subscales have been translated from Dutch to English by a certified translation agency with the original English FINC-NA terminology serving as a reference. They are expressed in truncated sentences to save space. Table 1 shows the homogeneity of the total FINC-FO scale with item-total correlations, internal consistency with the Cronbach’s alpha, and factor loadings per subscale.

The total FINC-FO questionnaire and the subscales *family as a resource in nursing care*, *family as a conversation partner*, and *family as its own resource* demonstrated strong internal consistency, with Cronbach’s alpha scores exceeding 0.80 across these scales. Most item-total correlations surpassed 0.40, with the exception of two items (RCN-1 and CP-5), which exhibited lower correlations. These two items also displayed inadequate factor loadings, below 0.40. Excluding them resulted in a slight improved Cronbach’s alpha. The Cronbach’s alpha for the subscale *family as a burden* was moderate, with one item showing a negative item-total correlation and the remaining items falling below 0.30.

Additionally, these FINC-FO score seem comparable to the Dutch FINC-NA questionnaire [16] which demonstrated similar reliability, with Cronbach’s alpha of 0.88 and 0.82 for the total score of the FINC-NA and subscale *family as a resource in nursing care*, respectively. However, the subscales *family as a conversational partner* and *family as its own resource* exhibited slightly lower Cronbach’s alpha values (0.74 and 0.73, respectively) compared to their counterparts in the FINC-FO. Conversely, the subscale *family as a burden* demonstrated slightly higher Cronbach’s alpha in the FINC-NA compared to the FINC-FO [16].

### Measuring families’ opinions

#### Study population

Table 2 illustrates the characteristics of the 1,275 respondents who completed the questionnaire. The average age of respondents was 60.7 years, and over 90% were between 40 and 80 years old. Over 70% were female, and more than half (57%) reported having paid employment. On average, these respondents worked 28 h a week, with 30% working 32 h a week or more. More than half of the respondents (59%) spent 8 h or less on caregiving tasks (ranging from 0 to 168 h), with 11.5% reporting spending at least 35 h on caregiving and 6.5% providing caregiving tasks 24 h a day.

**Table 1:** Results of the Families’ Importance in Nursing Care – Families’ Opinions (*n* = 1275)

	Percentages per category of response ^a^ (%)					Corrected item-Total correlation	α if item deleted	Factor Loading
Subscales	**1**	**2**	**3**	**4**	**5**			
Family as a resource in nursing care (RNC)								
1. Having a good relationship with nurses gives me a good feeling.	1	0	4	37	58	0.28	0.85	0.25
2. My presence when my family member receives care is meaningful.	4	10	33	32	22	0.50	0.82	0.73
3. My presence as a family member eases the workload of nurses.	4	13	40	30	13	0.56	0.81	0.78
4. It is important to me that I am invited to take an active part in the planning of care.	4	13	29	37	18	0.61	0.82	0.67
5. It is important to me that I am present when care is provided.	13	36	31	12	7	0.50	0.82	0.65
6. It is important to me to discuss show I can take an active part in care.	2	8	30	46	15	0.65	0.82	0.66
7. It gives me a feeling of being useful when I am involved in care.	4	12	32	40	12	0.60	0.82	0.66
8. I possess a lot of worthwhile knowledge about my family member that nurses can use in their work.	1	5	19	48	27	0.53	0.83	0.51
9. It is important to me that nurses spend time with me.	1	9	34	42	14	0.61	0.82	0.57
Cronbach’s alpha total subscale							0.84	
Family as a conversational partner (CP)								
1. It is important to me that nurses know who the patient’s family members are.	0	1	12	59	28	0.41	0.79	0.43
2. It is important to me that I am invited to take an active part in caring for my family member.	3	14	41	32	10	0.66	0.78	0.50
3. It is important to me that I am invited to a conversation at the start of care.	1	3	11	52	34	0.47	0.76	0.74
4. A conversation with me as a family member at the start of care will save nurses time in their work in the future.	1	4	22	48	24	0.58	0.76	0.74
5. The nurses found out who the family members are.	5	18	34	33	10	0.30	0.82	0.29
6. It is important to me that I am invited to a conversation at the end of care.	0	4	19	52	25	0.48	0.76	0.73
7. It is important to me that I am invited to a conversation when my family member’s situation changes or takes a turn for the worse.	0	1	5	50	45	0.48	0.77	0.65
8. It is important to me that I am regularly invited to a conversation on the progress (planning) of care.	1	5	27	46	22	0.59	0.76	0.67
Cronbach’s alpha total subscale							0.80	
Family as a burden (B) (recoded)								
1. I feel like I am holding nurses back in their work.	0	2	12	51	35	0.20	0.66	0.45
2. Nurses have no time to take care of me as a family member.	2	12	34	39	14	0.27	0.60	0.53
3. I feel like I should check on care, otherwise things will go wrong.	2	8	18	44	28	− 0.04	0.63	0.55
4. I get the impression that nurses feel stressed when I am present during care.	0	3	30	44	23	0.18	0.50	0.83
Cronbach’s alpha total subscale							0.67	
Family as its own resource (OR)								
1. It is important to me that nurses ask me how they can support me.	2	14	37	37	9	0.51	0.76	0.74
2. It is important to me that nurses encourage me to cope with the situation myself as best as I can.	4	15	45	31	5	0.51	0.74	0.78
3. It is important to me that nurses see me as a cooperating partner.	2	8	26	50	14	0.53	0.82	0.56
4. It is important to me that nurses help me cope with the situation as best as I can.	2	10	36	41	10	0.57	0.73	0.81
Cronbach’s alpha total subscale							0.81	
Cronbach’s alpha for the total FINC-FO							0.89	

^a^ 1 = Strongly disagree, 2 = Disagree, 3 = Neutral, 4 = Agree, 5 = Strongly agree

**Table 2:** Respondent characteristics (*n* = 1,275)

Family Characteristics		Mean	SD
Age (years)	Years	60.7	(10.9)
		**Median**	**25th and 75th percentile**
Caregiving hours (hours a week)	Hours	8.0	(4–14)
		**N**	**(%)**
Gender	Female	911	(71.6)
	Male	362	(28.4)
Education Level	High	521	(40.9)
	Middle	598	(46.9)
	Low	156	(12.2)
Relation to the patient	Spouse	327	(25.6)
	Parent/child	848	(66.5)
	Other	100	(7.9)
Paid Employment	Yes	731	(57.3)
	No	544	(42.7)

#### Scores on FINC-FO

The total score of 92.3 (SD 11.5; range 25–125), as well as the scores on the subscales of the FINC-FO questionnaire, represented approximately 70% of the maximum possible score (see Table 3). Table 1 illustrates the response percentages per category.

**Table 3:** Scores on the subscales of the Families’ Importance in Nursing Care- Families’ Opinions questionnaire(*n* = 1,275)

Subscales		Mean (SD)	Min–Max	Theoretical Range
Family as a resource in nursing care (RNC): 9 items		32.2 (5.7)	11**–**45	9**–**45
Family as a conversational partner (CP): 8 items		30.9 (4.2)	14**–**40	8**–**40
Family as a burden (B): 4 items		15.4 (2.5)	6**–**20	4**–**20
Family as its own resource (OR): 4 items		13.7 (2.8)	4**–**20	4**–**20
Total		**92.3 (11.5)**	**49–125**	**25–125**

#### Family as a resource in nursing care

Almost all respondents indicated that a good relationship with nurses gives them a positive feeling (95%), and most (75%) indicated having valuable knowledge that can be useful in caring for the patient or their family members. About half of the respondents indicated that their presence in care at home was meaningful (54%), made the work of a nurse easier (43%), and gave them a sense of purpose (52%). Family members also considered it important to actively participate in discussions about care (planning) and for nurses to allocate time for them. Fewer family members (19%) found it important to be present during actual care moments.

#### Family as a conversational partner

Of the family members, 87% found it important that nurses identify those who belong to the family, while less than half (43%) indicated that this had occurred in their situation. Most respondents (86%) considered it important to be invited for a conversation at the start of care provision, and 72% of respondents believed that this would save time. They also wished to be engaged in conversation at the end of care provision (77%), during changes (95%), or to regularly discuss progress (68%). Less than half (42%) found it important to be actively invited to participate in care provision.

#### Family as a burden

Most respondents (86%) did not believe that they hindered nurses in their work. Additionally, 67% did not feel that nurses found it difficult when family was present during care provision. Approximately 10% felt that they needed to monitor care provision to ensure that everything went well.

#### Family as its own resource

Nearly two-thirds (64%) of the respondents considered it important for nurses to view them as collaborative partners, while 10% did not find this important. Almost half of the respondents (46%) found it important to be asked how they could be supported, while 51% wanted support from nurses in coping with the situation. One-third (36%) found it important to be encouraged to cope with the situation as best as possible, while 19% did not find it important, and 45% had no opinion.

#### Differences according to background variables

Table 4 shows the scores for both the total FINC-FO questionnaire and the subscales related to the background variables.

#### Age groups

A significant difference was found in the scores between the age groups. Older (> 60 years) family members scored higher compared to younger family members on the overall FINC-FO and on the three subscales *family as a resource in nursing care*, *family as a burden*, and *family as its own resource* (*p* ≤ 0.005).

#### Gender

Gender showed no significant difference in scores, except on the subscale *family as a resource in nursing care.* On this subscale, male family members scored higher than female family members (*p* = 0.03).

#### Education level

Family members with low education levels showed a significant higher score on the overall FINC-FO compared to middle and high education levels (*p* < 0.001). This difference was also observed on the subscale *family as its own resource*. The subscale *family as a resource in nursing care* showed a statistically significant difference among family members of all education levels (*p* < 0.001).

#### Relationship to the patient

Spouses of patients scored significantly higher compared to other relationships on the total FINC-FO and on the three subscales *family as a resource in nursing care*, *family as a burden*, and *family as its own resource* (*p* < 0.001).

#### Paid employment

Family members who had paid employment scored significantly lower than family members who were unemployed or doing volunteer work on the total score of FINC-FO (*p* < 0.001) as well as on the three subscales *family as a resource in nursing care*, *family as a burden*, and *family as its own resource* (*p* ≤ 0.03).

#### Caregiving hours

The more care hours were provided by family members, the higher the scores on FINC-FO. Significant higher scores were seen in the total score of FINC-FO and on the subscales *family as a resource in nursing care*, *family as a conversational partner*, and *family as its own resource* (*p* < 0.001).

**Table 4:** Bivariate statistics between the Families’ Importance in Nursing Care- Families’ Opinion and background variables (*n* = 1,275)

Family Characteristics		Outcome FINC-FO Total		Family as resource in nursing care		Family as conversational partner		Family as burden		Family as its own resource	
		Mean (SD)	p-value	Mean (SD)	p-value	Mean (SD)	p-value	Mean (SD)	p-value	Mean (SD)	p-value
Age	≤ 60 year > 60 Year	91.1 (11.4) 93.5 (11.5)	< 0.001^***^	31.5 (5.7) 32.8 (5.5)	< 0.001^***^	30.8 (4.3) 31.0 (4.2)	0.39	15.2 (2.6) 15.6 (2.3)	0.004^**^	13.5 (2.8) 13.9 (2.9)	0.005^**^
Gender	Female Male	92.0 (11.0) 92.9 (12.6)	0.25	31.9 (5.6) 32.7 (5.9)	0.03^*^	31.0 (4.1) 30.9 (4.6)	0.76	15.4 (2.5) 15.4 (2.5)	0.84	13.7 (2.8) 13.8 (3.0)	0.39
Education Level	High Middle Low	91.0 (11.4) 92.3 (11.3) 96.0 (12.0)	< 0.001^***^	31.5 (5.7) 32.3 (5.5) 34.4 (5.6)	< 0.001^***^	30.9 (4.1) 30.9 (4.2) 31.4 (4.5)	0.43	15.3 (2.4) 15.5 (2.5) 15.6 (2.7)	0.18	13.4 (3.0) 13.7 (2.6) 14.7 (2.4)	< 0.001^***^
Relation	Spouse Parent - Child Other	96.5 (12.2) 90.7 (10.8) 91.8 (11.8)	< 0.001^***^	34.7 (5.7) 31.2 (5.4) 32.1 (5.6)	< 0.001^***^	31.2 (4.5) 30.8 (4.2) 31.1 (4.4)	0.55	16.0 (2.4) 15.2 (2.4) 15.0 (2.7)	< 0.001^***^	14.6 (2.7) 13.3 (2.8) 13.7 (3.2)	< 0.001^***^
Paid Employment	No Yes	94.3 (12.1) 90.7 (10.8)	< 0.001^***^	33.4 (5.7) 31.3 (5.4)	< 0.001^***^	31.2 (4.4) 30.8 (4.1)	0.12	15.6 (2.5) 15.3 (2.5)	0.03^*^	14.2 (2.8) 13.3 (2.8)	< 0.001^***^
Caregiving Hours (week)	0–8 9-168	89.5 (10.7) 96.8 (11.7)	< 0.001^***^	30.6 (5.3) 34.1 (5.6)	< 0.001^***^	30.4 (4.2) 31.8 (4.2)	< 0.001^***^	15.3 (2.5) 15.5 (2.5)	0.10	13.1 (2.8) 14.3 (2.9)	< 0.001^***^

^*^Significant *p* ≤ 0.05 ; ^**^Significant *p* ≤ 0.01; ^***^Significant *p* ≤ 0.001

#### Multiple linear regression

To determine the unique contribution of each background variable (see Table 4), multivariable linear regression models were performed for the FINC-FO questionnaire and its subscales (see Table 5). The number of caregiving hours made the greatest contribution for all subscales except *family as a burden*, and more caregiving hours resulted in a higher total FINC-FO score (β = 0.18; *p* < 0.001). The family relationship of spouses made the same contribution as caregiving hours on the subscale *family as a resource in nursing care* (β = 0.15; *p* < 0.001). Spouses made a significant contribution to the overall FINC-FO score (β = 0.09; *p* = 0.03), and on all subscales except *family as a conversational partner*. Low education level also contributed to the total FINC-FO score (β = 0.06; *p* = 0.04), as well as the subscales *family as a resource in nursing care* (β = 0.07; *p* = 0.02) and *family as its own resource* (β = 0.08; *p* = 0.02). Families with low education levels scored higher than those with middle education levels. For the subscale *family as a burden*, age made the greatest contribution (β = 0.10; *p* = 0.01).

Table 5 shows that only 2–9% is explained by the selected background variables.

**Table 5:** Multiple linear regression models of the Families’ Importance in Nursing Care–Families’ Opinion (*n* = 1,275)

	Total score of the FINC-FO (*R*² =0.073)		Family as resource in nursing care (*R*² =0.093)		Family as conversational partner (*R*² =0.022)		Family as a burden (*R*² =0.019)		Family as its own resource (*R*² =0.052)	
	β	p-value	β	p-value	β	p-value	β	p-value	β	p-value
Constant	-	< 0.001^***^	-	< 0.001^***^	-	< 0.001^***^	-	< 0.001^***^	-	< 0.001^***^
Age	− 0.01	0.75	− 0.02	0.58	− 0.03	0.46	0.10	0.01^**^	− 0.05	0.20
Gender^a^	0.04	0.25	0.03	0.41	0.02	0.46	0.03	0.39	0.04	0.26
Education level low^b^	0.06	0.04^*^	0.07	0.02^*^	0.04	0.27	− 0.02	0.49	0.08	0.02^*^
Education level high^b^	− 0.04	0.21	− 0.05	0.08	0.01	0.66	− 0.04	0.25	− 0.04	0.22
Relation spouse^c^	0.09	0.03^*^	0.15	< 0.001^***^	− 0.07	0.09	0.09	0.03^*^	0.08	0.05^*^
Relation other^c^	0.02	0.48	0.03	0.31	0.01	0.69	− 0.01	0.79	0.01	0.64
Paid Employment^d^	− 0.05	0.15	− 0.05	0.15	− 0.04	0.27	0.02	0.69	− 0.06	0.11
Caregiving Hours	0.18	< 0.001^***^	0.15	< 0.001^***^	0.18	< 0.001^***^	0.02	0.55	0.15	< 0.001^***^

^a^Reference = Male

^b^Reference = Education level middle

^c^Reference = Relation Parent-Child

^d^Reference = Paid Employment -No

^*^Significant *p* ≤ 0.05 ; ^**^Significant *p* ≤ 0.01; ^***^Significant *p* ≤ 0.001

## Discussion

In this study, we gained insight into families’ opinions regarding their involvement in nursing care at home using the developed FINC-FO questionnaire. The results specifically reveal that family members consider it important to be acknowledged as participants in discussions about care and care planning and that they wish for their knowledge and input to be appreciated. Family members seem less inclined to actively participate in care and express less explicit opinions about their own support needs. Overall, FINC-FO seems to be a feasible questionnaire to capture families’ opinions regarding their involvement in nursing care in the home setting.

Our study indicates that primarily, level of education, type of relationship, and amount of care provided are associated with opinions regarding involvement in care. In particular, spouses, family caregivers with a relative low level of education compared to middle and high level educated family members, and family caregivers providing more than eight hours of care express the wish to be involved in care for their relatives. As demonstrated in previous studies, the influences of these background characteristics often interconnect [20]. It seems obvious that spouses, who spend more time with patients, have the opportunity to provide more informal care. Also, people with lower resources in terms of education and income more often provide informal care because they are less inclined to utilize professional care and often have smaller social networks to assist with caregiving, and as a result, bear the burden of care themselves [21–24]. Healthcare professionals must be aware of these associations and the impact of these variables, as the desire for a high involvement in care and the inability to mobilize other resources to organize care might eventually contribute to the overloading of family members, which often happens gradually and when it becomes apparent it will become a crisis [25]. Prevention necessitates an approach that considers the entire care situation. Regular communication between patients, families, and healthcare professionals about collaborative caregiving and the division of roles and tasks is essential to ensure quality care in the long term and the sustainability of family caregiving.

Background variables discussed above explain only up to 9% of the variance regarding family involvement in care. This suggests that several other unidentified factors influence family members’ opinions and highlights the need for further research on this topic.

Although family members express a desire to be acknowledged as participants in discussions about care and care planning, they seem less inclined to be actively involved in actual care provision by nurses. Before healthcare professionals become involved, family members frequently perform myriad caregiving tasks. However, these responsibilities seem to shift when healthcare professionals become involved and take over the provision of care [26]. With the need for a transition from primarily formal care to a higher level of involvement of informal care, healthcare professionals should consider what care is already being provided by a family and discuss which additional aspects of care the family is willing and able to deliver by discussing the possibilities, wishes, and expectations in the provision of care with the family. This necessitates an awareness—on the part of both healthcare professionals and family members—of their respective roles and tasks in the provision of care [8]. In addition, it seems desirable that healthcare professionals and family members harmonize their principles, values, and mutual expectations regarding the provision of care for the patient. Such conversations will promote better collaboration and coordination based on mutual understanding.

Family members responded more neutrally on the subscale *family as its own resource*, which suggests that family members are focused primarily on the patient and less on themselves and their needs as family caregivers. Family members may be unaware of their need for support or expect nurses to be primarily dedicated to the patient, not to family members. However, from the perspective of family systems nursing, the focus of nurses should not be solely on the patient but on the care situation as a whole and the family as the unit of care [27]. Considering the transition from formal to more informal care, the awareness that families may need support seems relevant among healthcare professionals, and among the patients and families themselves [28].

### Strengths and limitations

The FINC-FO questionnaire was distributed via an electronic health record system, so it reached not the entire population of family members within the organization but only those who utilized the electronic patient portal. As a result, a possibility of bias in the results exists; family members who use the electronic patient portal may be more closely involved than those who do not. The sample size of 1275 allowed us to perform psychometric testing of the FINC-FO, indicating that the FINC-FO seems to be a feasible questionnaire to capture families’ opinions regarding their involvement in nursing care in the home setting. However, with a final response rate representing 35% of the total population, only cautious conclusions can be drawn about the population of family members of patients receiving homecare using the electronic patient portal. Further research is needed, employing alternative strategies to engage more respondents, in order to be able to generalize findings to a broader family population.

In this study, a questionnaire that has not yet demonstrated validity was employed. Nevertheless, the FINC-FO offer a sufficiently reliable and differentiated picture of family members’ opinions regarding their involvement in nursing care in the home setting, which suggests that this instrument can be recommend for use in future studies. However, it should be noted that the psychometric test conducted in this study indicates that the subscale *family as a burden* had moderate internal consistency as a subscale and a low item-total correlation with the total questionnaire. Depending on the primary questions in such studies, consideration may be given to adjusting or removing the subscale *family as a burden*; this domain seems to answer a nurse related topic as it concerns the perception of families toward nurses and is not related to family involvement. Therefore this subscale seems not to contribute meaningfully to the research question we posed as the starting point in our study. The internal consistency of the three other subscales had good reliability but could potentially be improved by removing two specific items (RNC-1: *Having a good relationship with nurses gives me a good feeling*; CP-5: *The nurses found out who the family members are*). These items also had the lowest factor loadings of the subscales, so removing or reformulating these items should be considered. RNC-1 seems to be more associated with generating positive emotions than functioning as a resource, while C-5 is not an opinion item. It asks about specific experiences, which does not fit in this questionnaire.

Further research will be needed to examine the performance of the FINC-FO questionnaire following further psychometric refinement and suitability in different (institutional) healthcare settings.

While many studies have investigated the perspective of nurses with regarding the role of family members in patient care, this study investigated how family members perceive their own role in patient care. Exploring how family members experience the involvement of nurses in the care for their loved-one, could also be an interesting lens to study in future research since nurses, at some point, enter the existing family system that initially takes up the care of the patient.

Despite the limit sample size in this study, it is vital to prioritize policy implications surrounding awareness among healthcare professionals and families regarding their caregiving roles. Interventions should be developed and implemented to enhance communication and fostering collaboration between healthcare providers and families. Healthcare education should emphasize the important of communication and implementation regarding the division of roles between nurses and family members in caregiving.

## Conclusion

In general, the family members of homecare patients want to be involved in nursing care. They wish to be acknowledged in discussions about care and care planning as participants with valuable knowledge. Family members are less inclined to actively participate in the care provided by nurses and are less explicit in their opinions about their own support needs.

The transition from primarily formal to more informal care necessitates an awareness on the part of both healthcare professionals and families of their respective roles in the provision of care. Communication about wishes, expectations, and the need for support in care is essential to ensuring quality care and that family members can sustain caregiving. With some suggestions for adjustment and improvement, FINC-FO is a feasible questionnaire to capture families’ opinions about their involvement in care.

## Data Availability

The FINC-FO questionnaire, meta data and The Strengthening the Reporting of Observational Studies in Epidemiology (STROBE) guidelines used to support the findings of this study has been deposited in the Dataverse repository. Available at : https://doi.org/10.34894/OYDOU4.

## Abbreviations

FINC-NA: Families’ Importance in Nursing Care–Nurses’ Attitudes
FINC-FO: Families’ Importance in Nursing Care–Families’ Opinions
RNC: Family as a resource in nursing care
CP: Family as a conversational partner
B: Family as a burden
CP: Family as a conversational partner

## References

[1] Jarling A, Rydström I, Fransson EI, Nyström M, Dalheim-Englund A, Ernsth Bravell M (2022). Relationships first: formal and informal home care of older adults in Sweden. Health Soc Care Community.

[2] Årestedt L, Persson C, Benzein E (2014). Living as a family in the midst of chronic illness. Scand J Caring Sci.

[3] Rolland JS (2017). Neurocognitive impairment: addressing couple and Family challenges. Fam Proc.

[4] Haley WE, Roth DL, Sheehan OC, Rhodes JD, Huang J, Blinka MD (2020). Effects of transitions to Family Caregiving on Well-Being: a Longitudinal Population-based study. J Am Geriatr Soc.

[5] Roth DL, Fredman L, Haley WE (2015). Informal caregiving and its impact on health: a reappraisal from population-based studies. Gerontologist.

[6] Johnsson A, Wagman P, Boman Å, Pennbrant S (2019). Striving to establish a care relationship-mission possible or impossible?-Triad encounters between patients, relatives and nurses. Health Expect.

[7] Zotterman AN, Skär L, Söderberg S (2018). Meanings of encounters for close relatives of people with a long-term illness within a primary healthcare setting. Prim Health Care Res Dev.

[8] Hagedoorn EI, Paans W, Jaarsma T, Keers JC, van der Schans CP, Luttik MLA (2021). The importance of families in nursing care: attitudes of nurses in the Netherlands. Scand J Caring Sci.

[9] Bei E, Zarzycki M, Morrison V, Vilchinsky N (2021). Motivations and willingness to provide care from a geographical distance, and the impact of distance care on caregivers’ mental and physical health: a mixed-method systematic review protocol. BMJ Open.

[10] de Klerk M, de Boer A, Plaisier I (2021). Determinants of informal care-giving in various social relationships in the Netherlands. Health Soc Care Community.

[11] Zarzycki M, Seddon D, Bei E, Dekel R, Morrison V (2022). How Culture shapes Informal Caregiver motivations: a Meta-Ethnographic Review. Qual Health Res.

[12] Woldring J, Paans W, Gans R, Dorland L, Luttik ML. Measuring the opinions of family members about their involvement in care (dataset). 2023; 10.34894/OYDOU4.

[13] Saveman B, Benzein EG, Engström ÅH, Årestedt K (2011). Refinement and psychometric reevaluation of the instrument: families’ importance in nursing care–nurses’ attitudes. J Fam Nurs.

[14] Benzein E, Johansson P, Arestedt KF, Berg A, Saveman B (2008). Families’ importance in nursing care: nurses’ attitudes–an instrument development. J Fam Nurs.

[15] Alfaro Díaz C, Esandi Larramendi N, Gutiérrez-Alemán T, Canga-Armayor A (2019). Systematic review of measurement properties of instruments assessing nurses’ attitudes towards the importance of involving families in their clinical practice. J Adv Nurs.

[16] Hagedoorn EI, Paans W, Jaarsma T, Keers JC, van der Schans CP, Luttik ML (2018). Translation and psychometric evaluation of the Dutch families importance in nursing care: nurses’ attitudes Scale based on the generalized partial credit Model. J Fam Nurs.

[17] Benzein E, Johansson P, Arestedt KF, Saveman B (2008). Nurses’ attitudes about the importance of families in nursing care: a survey of Swedish nurses. J Fam Nurs.

[18] Field A. Discovering statistics using IBM SPSS statistics. 4th ed. SAGE; 2013.

[19] Stevens JP (2002). Applied multivariate statistics for the social sciences.

[20] Hengelaar AH, Wittenberg Y, Kwekkeboom R, Van Hartingsveldt M, Verdonk P (2023). Intersectionality in informal care research: a scoping review. Scand J Public Health.

[21] Quashie NT, Wagner M, Verbakel E, Deindl C (2022). Socioeconomic differences in informal caregiving in Europe. Eur J Ageing.

[22] Bertogg A, Strauss S (2020). Spousal care-giving arrangements in Europe. The role of gender, socio-economic status and the welfare state. Ageing Soc.

[23] Schmitz A, Quashie NT, Wagner M, Kaschowitz J. Inequalities in caregiving strain during the COVID-19 pandemic: conceptual framework and review of the empirical evidence. Int J Care Caring 2022:1–14.

[24] Abbing J, Suanet B, van Groenou MB (2023). Socio-economic inequality in long-term care: a comparison of three time periods in the Netherlands. Ageing Soc.

[25] Baptista BO, Beuter M, Girardon-Perlini NMO, Brondanid CM, de Budó MLD, dos Santos NO (2012). Overload of family caregiver at home: an integrative literature review. Rev Gaucha Enferm.

[26] Lindahl B, Lidén E, Lindblad B (2011). A meta-synthesis describing the relationships between patients, informal caregivers and health professionals in home-care settings. J Clin Nurs.

[27] Wright L, Leahey M (2019). Nurses and families: a guide to Family Assessment and intervention.

[28] Park M, Giap T, Lee M, Jeong H, Jeong M, Go Y (2018). Patient- and family-centered care interventions for improving the quality of health care: a review of systematic reviews. Int J Nurs Stud.

